# Safety and effectiveness of fondaparinux as a postpartum thromboprophylaxis during puerperium among muslim women: A single centre prospective study

**DOI:** 10.3389/fphar.2022.887020

**Published:** 2022-09-23

**Authors:** Normaliza Muhamad, Muhammad Azrai Abu, Aida Hani Kalok, Mohd Nasir Shafiee, Shamsul Azhar Shah, Nor Azlin Mohamed Ismail

**Affiliations:** ^1^ Department of Obstetrics and Gynecology, UKM Medical Centre, Kuala Lumpur, Malaysia; ^2^ Department of Public Health, UKM Medical Centre, Kuala Lumpur, Malaysia

**Keywords:** fondaparinux, postpartum, thromboprophylaxis, pulmonary embolism, muslim women

## Abstract

**Background:** Venous thromboembolism (VTE) remains one of the leading causes of maternal morbidity and mortality, with postpartum period carrying the greatest risk. Perinatal thromboprophylaxis is often administered based on risk-factor assessment. Low molecular weight heparin has a proven safety profile in the obstetrics population, however, its porcine-derived content may lead to reduced uptake amongst certain religious groups. We aimed to evaluate the safety of fondaparinux as an alternative postpartum thromboprophylaxis.

**Methods:** We conducted a prospective, single arm, open label study from September 2017 until March 2018. Women who fulfilled the criteria for post natal thromboprophylaxis based on the 2015 RCOG guidelines were recruited. Each patient received subcutaneous injection of Fondaparinux, 2.5 mg daily for 10 days. A telephone interview was conducted on day 10 post delivery. Each woman was subsequently reviewed in the outpatient clinic 6 weeks postpartum. The primary outcome measure was occurrence of pulmonary embolism or deep vein thrombosis suggestive by clinical symptoms and assessment. Secondary outcome measures were allergic reaction and bleeding tendency such as secondary post-partum haemorrhage, spinal site bleeding and wound haematoma. Allergic reaction and bleeding tendency in neonates were also recorded.

**Results:** Sixty women were included in the analysis. There were no VTE cases amongst our cohort. No major bleeding was recorded. Two patients (3.3%) had wound haematoma, one of which occurred 3 weeks post delivery. No adverse effect in neonates was noted.

**Conclusion:** Fondaparinux is a safe alternative thromboprophylaxis for postpartum women.

## Introduction

Venous thromboembolism (VTE) remains amongst the major causes of maternal morbidity and mortality in developed countries (Devis and Knuttinen, 2017; Merriam et al., 2018). Women are five times more likely to develop this condition in pregnancy (Devis and Knuttinen, 2017), with the greatest risk occurring during postpartum period (Abdul Sultan et al., 2013). Hypercoagulability, venous stasis due to vena cava compression and decreased mobility in pregnancy, are all contributing to the pathophysiology of VTE(2).

Inherited or acquired thrombophilia, a previous history of thrombosis, antiphospholipid syndrome, lupus, heart disease and sickle cell disease are significant risk factors for VTE in pregnancy, which may require antenatal thromboprophylaxis (James et al., 2006). Factors such as maternal age above 35, nulliparity, multiple pregnancy and smoking are associated with 1.5 to 2 fold-elevated risk. The VTE risk amongst obese pregnant mothers is four times higher than those with normal BMI whilst caesarean delivery increases the risk by two to five-fold (James et al., 2006; Jacobsen et al., 2008; Thurn et al., 2018). Thurn et al., demonstrated that women with preeclampsia were at a threefold higher risk postpartum, with no significant increased risk before delivery (Thurn et al., 2018).

Clinical signs and symptoms of VTE such as dyspnoea, reduced effort tolerance, leg pain and swelling have considerable overlap with normal pregnancy-related physiologic adaptations, which pose a diagnostic dilemma. The use of imaging is central to the diagnosis of VTE. Compression duplex ultrasonography (CUS) with the addition of color flow doppler is the first-line imaging technique to confirm the suspected DVT in pregnancy. In suspected PE cases, both ventilation/perfusion (V/Q) scans and computed tomographic pulmonary angiography (CTPA) studies are appropriate imaging options (Mc and James, 2018), which carry minimal radiation risk exposure to both mother and fetus.

Low Molecular Weight Heparin (LMWH) represents the most efficacious and safe anticoagulant during pregnancy. It does not cross the placenta like warfarin and is associated with lower rate of bleeding complications than that of the unfractionated heparin. LMWH was also demonstrated to be minimally secreted in breast milk (Greer and Nelson-Piercy, 2005). The drug is porcine based with longer half-life and increased bioavailability, hence the once daily dose of administration via subcutaneous injection (White, 2006). The drug clearance is dose dependent and more predictable in comparison to heparin thus eliminates the need for coagulation profile monitoring.

Fondaparinux Sodium (Arixtra^®^) is a synthetic selective inhibitor of activated factor X (FXa). At present, there is insufficient data to support routine use of fondaparinux in pregnancy except for cases such as heparin-induced thrombocytopenia or severe allergy to heparin (Dempfle, 2004; Knol et al., 2010). Fondaparinux has been extensively studied as surgical thromboprophylaxis, however research on its role as in postpartum VTE prevention has been limited to retrospective studies (Kawaguchi et al., 2017; Kumar et al., 2019).

Malaysia has a multi-ethnic population with Islam as the official religion. A retrospective study on postpartum thromboprophylaxis in a multi-religious Malaysian cohort, demonstrated that almost a third of muslim women opted for unfractionated heparin, citing concerns over the porcine derivative of LMWH (Voon et al., 2018). Although muslims are allowed receive drug of porcine origin when there is risk to life and safe alternative is unavailable, patients ultimately have the right to choose (White, 2006). A safety study on fondaparinux is therefore essential in the effort to provide women with different treatment option for VTE prophylaxis. We aimed to evaluate the safety profile of fondaparinux as thromboprophylaxis in postpartum women.

## Material and method

We conducted a single arm, open label, prospective study over six-month period, from September 2017 to March 2018. Prior approval from the Universiti Kebangsaan Malaysia Research and Ethics Board (Research Code: FF-2017-450) was obtained. Post-partum Malaysian women above the age of eighteen, who scored as intermediate risk based on the RCOG VTE risk assessment (Royal College of Obstetricians and Gynaecologists, 2015), were recruited and written consent was obtained. Exclusion criteria were history of hypersensitivity to Fondaparinux, bleeding disorder, weight less than 50 kg or more than 90 kg, hypertensive disorder and classified as high risk on RCOG criteria.

All patients were started on subcutaneous Fondaparinux Sodium (Arixtra^®^ Aspen, Noter Dame de Bondleville, France) around 6 hours post-delivery. The administered dosage was 2.5 mg daily for 10 days. All women were taught the injection technique and were counselled regarding the symptoms of bleeding and VTE, prior to discharge. All patients were advised to wear compression stockings and ensure adequate hydration at home. We conducted phone interview on each participant after the completion of the 10-day thromboprophylaxis course. All women were reviewed by the investigator at 6 weeks post-partum in clinic, for clinical evidence of DVT or PE. The primary outcome measure were the occurrence of DVT or PE, evidenced by clinical symptoms such as leg swelling, chest pain, shortness of breath or difficulty breathing. Patients were advised to come to hospital for assessment and confirmatory test, if they developed any of the symptoms during the post-natal period. The secondary outcome measures were allergic reaction and bleeding tendency such as secondary post-partum haemorrhage, spinal site bleeding, wound haematoma. Any bleeding tendency and allergic reaction in neonates were also recorded.

Sample size estimation was calculated using the population proportion formula (Lemeshow, Hosmer, Klar, Lwanga, and Organization, 1990). Prior data indicated that the prevalence of DVT amongst women who received enoxaparin as thromboprophylaxis after caesarean section, was 3.9% (Goto et al., 2015). Assuming a precision of 5% with power study of 0.8 and dropout rate of 30%, the minimum sample size required for our study was 59. Data were recorded and analysed using Statistical Package for Social Science (IBM SPSS Statistics, Version 24.0 Armonk, NY: IBM Corp). The demographics and outcome were analysed using descriptive statistics.

## Results

There were 2,653 deliveries during the study period with 60 patients who fulfilled recruitment criteria. Table 1 demonstrates the women’s demographics alongside delivery details. The mean age and BMI were 33.2 and 29.7 kg/m^2^ respectively. Majority of our cohort (95%) underwent caesarean sections whilst the rest had vaginal births. 93.3% of our subjects had regional anesthesia, in form of epidural and spinal anaesthesia and only four women underwent general anaesthesia. Around 63.3% of caesarean sections were for maternal indications whilst the remaining cases were delivered due to fetal reasons. Our cohort compliance rate was 93.3%.

**TABLE 1:** Baseline characteristic of patients.

Demographic data	Frequency *n* = 60
Maternal age (years)	
Mean ± s.d	33.2 ± 4.1
Maternal age group (years)	
19–34	39 (65%)
35—45	17 (28.3%)
>45	4 (6.7%)
Race	
Malay	58 (96.6%)
Indian	2 (3.4%)
Parity	
Primipara	1 (1.7%)
Multipara	59 (98.3%)
Booking BMI (kg/m2)	
Mean ± sd	29.7 ± 3.9
Class of obesity	
Normal	9 (15%)
Class 1	16 (26.7%)
Class 2	29 (48.3%)
Class 3	6 (10%)
Mode of delivery	
Caesarean section	57 (95%)
Spontaneous vertex delivery	2 (3.3%)
Instrumental delivery	1 (1.7%)
Anesthesia	54 (90%)
Regional	
Spinal	
Epidural	2 (3.3%)
General	4 (6.7%)
Compliance	
Yes	56 (93.3%)
No	4 (6.7%)

None of our subjects reported clinical symptoms of VTE at day 10 post-partum. All except two women turned up for six-week outpatient assessment. None of the patients demonstrated clinical evidence of DVT or PE throughout the study duration. There were no reported allergic reaction, secondary postpartum haemorrhage or epidural/spinal site bleeding. Allergic reaction and bleeding tendency were also absent amongst the neonates.

Wound hematoma occurred in two women (3.3%). The first subject developed wound haematoma over her pfannenstiel incision on day 3 post-delivery. She underwent caesarean section for severe preeclampsia with a background of gestational diabetes mellitus and two previous caesarean sections. There was no evidence of thrombocytopenia or prolonged coagulation profile. Fondaparinux was stopped from day three and the haematoma subsequently resolved with conservative management. Another patient developed superficial caesarean wound hematoma around day 30 postpartum. She did not report the finding until she attended the six-week postnatal assessment. Patient underwent caesarean section for fetal distress and her antenatal risk factor was obesity with booking BMI of 35.9 kg/m^2^. Upon assessment in clinic, the wound hematoma had resolved, with no evidence of other bleeding tendency.

## Discussion

To our knowledge, this is the first prospective study to evaluate the safety of fondaparinux as post-partum thromboprophylaxis. In this study, we assessed the effect of Fondaparinux in postpartum patients who had moderate risks of VTE. The patients recruited in our study represented our patients in daily practice. We did not find any evidence of clinical VTE in our cohort. There was no major bleeding which required hospitalisation. Two women had wound haematoma, one of which occurred almost 3 weeks after the completion of fondaparinux. Taking this into account, the prevalence of bleeding which could be attributed to the thromboprophylaxis was only 1.7%. A retrospective study from Japan, demonstrated that the rate of major bleeding complication was 0.68% whilst wound hematoma and superficial bleeding occurred in six (2.03%) of 295 patients. Kawaguchi et al. reported zero incidence of symptomatic VTE or any VTE related maternal death in their cohort of 295 women who received fondaparinux as prophylaxis following caesarean delivery (Kawaguchi et al., 2017). A recent meta-analysis comparing fondaparinux with LMWH for perioperative surgical thromboprophylaxis, concluded that the former demonstrated superior efficacy in VTE reduction but was associated with greater odds of major bleeding. Pooled analysis of 12 randomised control trials involving 14,906 patients found that the odds of VTE in the fondaparinux group were 0.49 times the odds in LMWH group (Odd ratio, OR = 0.49; 95% confidence interval, CI, 0.38–0.64; *p* < 0.001). Kumar et al. found that fondaparinux was associated with increased risk of major bleeding, especially involving surgical site (OR = 1.48; 95% CI, 1.15–1.90; *p* = 0.002) (Kumar et al., 2019). Although the result of meta- analysis suggested a trade-off between safety and efficacy when fondaparinux was used over LMWH; the authors discovered that net clinical benefit was in favour of fondaparinux compared to the latter. The observed differences in the efficacy and safety of both drugs may be explained by their pharmacokinetics. Fondaparinux specific anti-Xa activity is higher than that of LMWH (about 700 units/mg and 100 units/mg, respectively), whilst its half-life after subcutaneous injection is longer than that of LMWH (around 17 and 4 h, respectively) (Garcia et al., 2012).

At present, there are no published data on the excretion of fondaparinux into breast milk. Fondaparinux is a synthetic analog of the anti thrombin-binding pentasaccharide found in heparin and LMWH (Garcia et al., 2012). Previous study on LMWH in lactating women concluded that significant absorption by breastfeeding infants is unlikely, as orally ingested heparins have low bioavailability (Richter et al., 2001). This led to recommendation by the American Society of Haematology in 2018, on the safe use of fondaparinux in women whom anticoagulation is indicated (Bates et al., 2018). Our subjects did not report any bruising or bleeding tendencies in their babies, throughout the study period.

Post-caesarean thromboprophylaxis has been identified as a mean of systemically reducing VTE-related maternal mortality (Clark et al., 2008). Decision and economic analyses also support the use of universal mechanical thromboembolism prophylaxis following caesarean section (Palmerola et al., 2016; Lamont et al., 2019). The guidelines for the pharmacologic thromboprophylaxis however, differ substantially between major societies such as the American Congress of Obstetricians (ACOG), the British Royal College of Obstetricians and Gynaecologists (RCOG), and the American College of Chest Physicians (Chest). Study by Palmerola et al. demonstrated that the percentage of women who would receive unfractionated heparin (UFH) or LMWH following caesarean section based on the ACOG and Chest guidelines were around 1 and 35% respectively. RCOG risk factor-based approach is the most conservative and its recommendations resulted in 80–90% patient coverage (Quiñones et al., 2005; Royal College of Obstetricians and Gynaecologists, 2015). RCOG risk assessment tool has not been validated in terms of clinical and cost effectiveness, as it would be difficult to conduct clinical trial given the VTE incidence rate and very large sample size requirement (Lamont et al., 2019). RCOG recommends the use of LMWH for thromboprophylaxis. Unfractionated heparin is advocated in cases with very high thrombotic risk, whilst fondaparinux is reserved for women who are intolerant of heparin compounds (Royal College of Obstetricians and Gynaecologists, 2015). Data from the UK and Sweden indicated that Fondaparinux is more cost effective than enoxaparin as thromboprophylaxis in major orthopaedic surgery (Gordois et al., 2003; Lundkvist et al., 2003). Currently, there is no cost-analysis data of fondaparinux use in the obstetrics population.

Animal derived products use for medical and surgical treatment may conflict with patients’ religious beliefs. A survey conducted by Eriksson et al. amongst religious and spiritual leaders of six largest religions worldwide, reported that porcine containing products were forbidden not only for the Muslims, but also Hindus and Sikhs. Bovine derived materials were not permissible amongst the Hindus and Sikhs. However, all religions accepted the use of all these products in case of an emergency and only if alternatives were not available (Eriksson et al., 2013). The Malaysia National Fatwa Council in 2009 had made a ruling that the use of porcine-based clexane and fraxiparine is prohibited unless there are no other halal alternatives. Retrospective study on postnatal thromboprophylaxis amongst Malaysian women revealed that the uptake was remarkably high (99.7%) indicating high level of trust towards healthcare providers and awareness from antenatal and preoperative counselling (Voon et al., 2018). However, only 70% of Muslim women opted for the recommended LMWH, which most likely related to its animal derived content. Women have the rights to choose and give consent over the treatment they receive; hence it is crucial to prove the safety profile of other alternative thromboprophylaxis.

Despite the small sample size, our study showed promising evidence of the clinical safety of fondaparinux as postpartum VTE prophylaxis in a short duration. Further larger clinical trial with involvement of high-risk group of VTE is required to understand the impact of Fondaparinux among postpartum patients. Future research to evaluate the efficacy of the drug against enoxaparin, through randomised controlled trial, using a diagnostic imaging study, with longer follow up period is essential to further validate its use as safe and effective alternative to LMWH.

In conclusion, fondaparinux appears safe in preventing VTE during the postpartum period. Although no cases of breakthrough VTE were recorded in our cohort, further large studies are required to confirm this outcome. Thorough assessment of patients from antenatal until postpartum is crucial to identify risk of VTE.

## Data Availability

The raw data supporting the conclusions of this article will be made available by the authors, without undue reservation.
